# Poultry Infection with Influenza Viruses of Wild Bird Origin, China, 2016

**DOI:** 10.3201/eid2407.171220

**Published:** 2018-07

**Authors:** Zhijun Yu, Kaihui Cheng, Yuwei Gao

**Affiliations:** Institute of Poultry Science of Shandong Academy of Agricultural Sciences, Jinan, China (Z. Yu);; Shandong Academy of Agricultural Sciences, Jinan (Z. Yu, K. Cheng);; Dairy Cattle Research Center of Shandong Academy of Agricultural Sciences, Jinan (K. Cheng);; Military Veterinary Research Institute of Academy of Military Medical Sciences, Changchun, China (Y. Gao)

**Keywords:** Migratory birds, domestic poultry, transmission, H13N2, H13N8, viruses, influenza, influenza virus, public health, zoonoses, China

## Abstract

Migratory birds may play a role in transmission of avian influenza virus. We report the infection of black-tailed gulls and chickens in eastern China with avian influenza (H13N2) and (H13N8) viruses. We found that these H13 viruses were transmitted from migratory birds to domestic poultry.

Avian influenza virus with 10 hemagglutinin (HA) subtypes has emerged in poultry (*1*), and the potential role of migratory birds in transmission of avian influenza virus has caused concern (*2*). We report infection with low pathogenicity avian influenza (LPAI) virus of HA subtype 13 (H13) among migratory birds (black-tailed gulls [*Larus crassirostris*]) and domestic poultry (chickens) in Weihai, Shandong Province, eastern China (Technical Appendix Figure 1).

Weihai is a breeding center for black-tailed gulls that congregate from northern Asia, eastern Asia, Southeast Asia, and North America. These gulls reside along the coastlines of the East China Sea and Japan and have been found as vagrants in Alaska, North America, and the Philippines (*3*). In China, black-tailed gulls perch at the Longxudao wharf (N37°23′24.05′′, E122°41′26.16′), located in the northeastern corner of Weihai. In December 2016, we collected 149 fecal samples from black-tailed gulls at Longxudao wharf and screened them for evidence of influenza virus by reverse transcription PCR, DNA sequencing, and BLAST (http://www.ncbi.nlm.nih.gov/blast/Blast.cgi) analysis in the GenBank database. After independently inoculating positive fecal samples into the allantoic cavities of specific pathogen-free embryonated chicken eggs, we obtained 6 influenza H13N2 and 60 influenza H13N8 virus isolates.

To assess the epidemiologic characteristics of these H13 isolates, we completely sequenced an H13N2 isolate (A/black-tailed gull/Weihai/115/2016) and an H13N8 isolate (A/black-tailed gull/Weihai/17/2016) (GenBank accession nos. MF461177–92). Phylogenetic analysis indicated that their HA and neuraminidase (NA) segments were derived from the Eurasian lineage, in accordance with their geographic distribution (Technical Appendix Figures 2, 3). Moreover, the H13N2 and H13N8 isolates possessed high nucleotide sequence identity to the avian influenza virus subtypes previously isolated from Europe, Asia, and North America (Technical Appendix Table 1). We speculate that avian influenza virus subtypes H13N2 and H13N8 are reassortants between the Eurasian and North American lineages (Technical Appendix Figures 4, 5).

We next analyzed the timing of the reassortment events that led to the emergence of subtype H13N2 (Figure, panel A). During July 2009, June 2012, July 2009, and June 2015, the following genes, respectively, were transferred from seagulls in Europe: HA, nucleocapsid protein (NP), matrix (M), and nonstructural (NS). During 2004, November 2011, and October 2014, the following genes, respectively, originated from waterfowl in Asia: polymerase basic (PB) 1, polymerase acidic (PA), and NA. In November 2007, the PB2 gene was transferred from avian influenza viruses circulating among wild waterfowl in North America. 

We also estimated the timing of the reassortment events that led to the emergence of subtype H13N8 (Figure, panel B). During June 2012, July 2013, July 2013, and June 2015, the following genes, respectively, were transferred from seagulls in Europe: NP, NA, M, and NS. During 2004, November 2011, and September 2012, the following genes, respectively, originated from waterfowl in Asia: PB1, PA, and HA. In November 2007, the PB2 gene was transferred from avian influenza viruses circulating among wild waterfowl in North America. 

**Figure:**
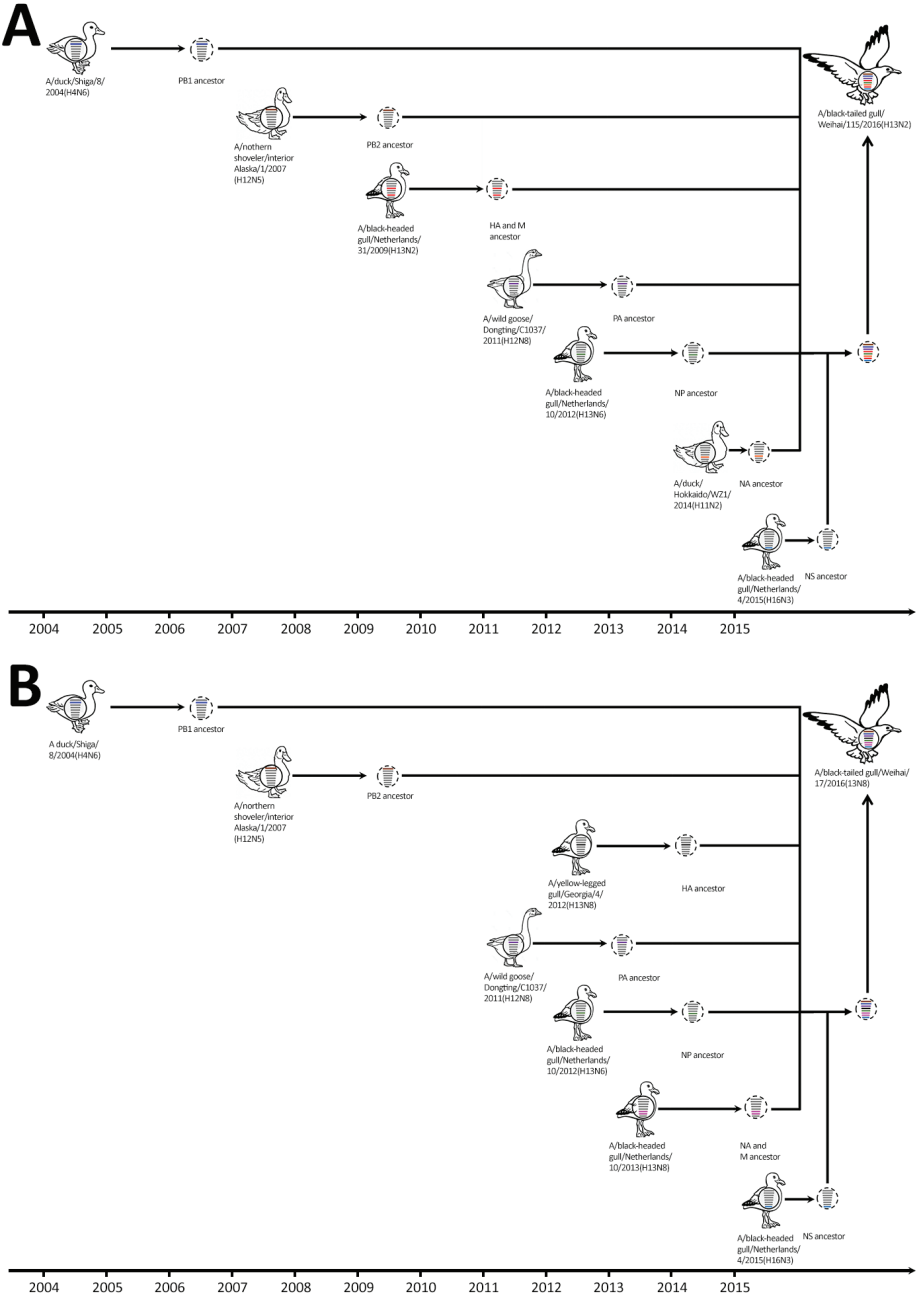
Hypothetical evolutionary pathway of avian inﬂuenza viruses of H13N2 (A) and H13N8 virus (B) subtypes isolated from black-tailed gulls in eastern China, 2016. Gene segments are colored according to their origin. Dashed virions indicate unidentified viruses. HA, hemagglutinin; M, matrix protein; NA, neuraminidase; NP, nucleoprotein; NS, nonstructural protein; PA, polymerase acidic protein; PB, polymerase basic protein.

According to these data, the generation of influenza virus subtypes H13N2 and H13N8 in seagulls seems to have been a complex process and was probably completed in the middle of 2015 (Figure). We also found that subtypes H13N2 and H13N8 possessed some molecular markers associated with increased virulence and transmission among mammals (Technical Appendix Table 2).

In April 2017, we analyzed serum samples collected from 48 chickens at a chicken farm at Songcun town (N37°04′39.96′′, E122°00′38.83′′) in Weihai for serologic evidence of exposure to H13 viruses. We found detectable hemagglutinin inhibition (HI) antibody titers against H13N2 virus in 4 (8.3%) samples and detectable HI antibody titers against H13N8 virus in 14 (29.2%) samples (Technical Appendix Table 3). When we evaluated reference serum samples known to contain HI antibodies against each of the virus subtypes for potential cross-reactivity, we observed no apparent cross-reactivity of H13 antibodies against 7 other HA subtype viruses (Technical Appendix Table 6). Therefore, although the serum samples’ HI antibody titers against H13 viruses were not high, we cannot exclude the possibility that these antibodies were generated in response to independent exposure to H13 viruses.

In March 2013, the novel LPAI H7N9 virus causing serious human infections was detected in eastern China (*4*–*7*); after circulating among domestic poultry, this virus evolved into a highly pathogenic virus (*8*). Therefore, enhanced surveillance is needed to determine whether other LPAI viruses could be introduced into domestic poultry and pose a threat to public health. 

In this study, we isolated a large number of LPAI H13 viruses from seagulls at the Longxudao wharf and detected H13-specific seroconversion in chickens at a chicken farm, which is ≈100 km west of this wharf and lies on the migratory route of black-tailed gulls. These findings indicate that H13 viruses may have been introduced into domestic poultry from migratory birds and that they may have the potential to become a global cross-species threat. 

Technical Appendix Additional methods and results for study of poultry infection with influenza viruses of wild bird origin, China, 2016.
