# Supplementary material for: Poultry Infection with Influenza Viruses of Wild Bird Origin, China, 2016
Source: Emerg Infect Dis. 2018 Jul;24(7):1375–7. doi: 10.3201/eid2407.171220 (PMC6038736; doi:10.3201/eid2407.171220)
Supplement: Technical Appendix — Additional methods and results for study of poultry infection with influenza viruses of wild bird origin, China, 2016. [file 17-1220-Techapp-s1.pdf]

Article DOI: https://doi.org/10.3201/eid2407.171220

Poultry Infection with Influenza Viruses of Wild Bird Origin, China, 2016

Technical Appendix

Detailed Methods

Sample Collection

In the northeast corner of Weihai City is located the Longxudao wharf (GPS position: N37°23′24.05,” E122°41′26.16”) which a large number of black-tailed gulls perch at. A lot of trawlers moored alongside the longxudao wharf after the fishing, and a small amount of seafood was left at the wharf after the unloading process of the trawlers. The black-tailed gulls ate the seafood, and the feces of these seabirds were left at the longxudao wharf. In December 2016, we picked up one hundred and forty-nine feces specimens of black-tailed gulls at the Longxudao wharf.

RNA Isolation, RT-PCR Amplification, Sequencing, BLAST, and Virus Isolation

All one hundred and forty-nine feces specimens of black-tailed gulls were homogenized in 800μl PBS supplemented with 2000 IU/ml penicillin and 2000 mg/ml streptomycin. The disrupted feces specimens were centrifuged to remove debris and the feces supernatants were used for further study. We screened all feces supernatants by RT-PCR, sequencing, and BLAST for evidence of influenza virus infection. Viral RNA was isolated from the feces supernatants using the RNeasy Mini kit (QIAGEN, Germantown, MD) according to the manufacturer's protocol. Reverse transcription of viral RNA and subsequent PCR were performed using primers specific for H13, H16, N2, N3, N6, and N8 (Technical Appendix Table 4). All positive PCR products were purified using the QIAquick PCR purification kit (QIAGEN, Germantown, MD) according to the manufacturer's protocol and were sequenced by the Tsingke Genomics Institute (Qingdao, China). Sanger sequencing methodology was used to sequence the PCR-amplified viral gene segments. DNA sequences were analyzed using the Lasergene sequence analysis software package (DNASTAR, Madison, WI). There are sixty feces supernatants identified as

H13N8 positive by RT-PCR, subsequent DNA sequencing, and BLAST analysis in the GenBank database (GenBank database URL: https://blast.ncbi.nlm.nih.gov/Blast.cgi?PROGRAM = blastn&PAGE_TYPE = BlastSearch&LINK_LOC = blasthome). Moreover, there are other six feces supernatants identified as H13N2 positive by RT-PCR, subsequent DNA sequencing, and BLAST analysis in the GenBank database. Furthermore, all sixty-six AIV-positive feces supernatants were independently inoculated in the allantoic cavities of 10-day-old specific pathogen-free embryonated chicken eggs to isolate viruses. After 72-hour incubation at 37°C, we recovered six H13N2 virus isolates and sixty H13N8 virus isolates. Additionally, serum samples collected from 48 chickens residing in a chicken farm at Songcun town (GPS position: N37°04′39.96″, E122°00′38.83″) in Weihai city were analyzed for serologic evidence of H13N2 and H13N8 AIV exposure.

Genome Sequencing

The viral RNA of A/black-tailed gull/Weihai/115/2016 (H13N2) or A/black-tailed gull/Weihai/17/2016 (H13N8) was independently isolated from the allantoic fluid of inoculated eggs using the RNeasy Mini kit (QIAGEN, Germantown, MD) according to the manufacturer's protocol. Reverse transcription of viral RNA and subsequent PCR were performed using primers specific for each gene segment (Technical Appendix Table 4 and Technical Appendix Table 5). PCR products were purified using the QIAquick PCR purification kit (QIAGEN, Germantown, MD) according to the manufacturer's protocol. Viral gene segments were sequenced by the Tsingke Genomics Institute (Qingdao, China). The GenBank accession numbers of A/black-tailed gull/Weihai/115/2016 (H13N2) and A/black-tailed gull/Weihai/17/2016 (H13N8) are MF461177 to MF461192.

Phylogenetic Analysis

To investigate the molecular and epidemiologic characteristics and to determine the profile of genetic diversity, phylogenetic trees were constructed using molecular evolutionary genetics analysis MEGA 7 (http://www.megasoftware.net/mega.php) with the neighbor-joining (NJ) method to calculate distance.

Hemagglutination Inhibition (HI) Assay

HI tests were performed as previously described (*1*,*2*). Briefly, serum samples were treated with receptor-destroying enzyme (RDE; Sigma-Aldrich, St. Louis, MO, USA; 1 part

serum:4 parts RDE) overnight at 37°C to remove nonspecific inhibitors before analysis. Five parts of 1.5% sodium citrate were added and the samples heat-inactivated at 56°C for 30 min. Serum samples were then serially diluted 2-fold in phosphate buffered saline (PBS). Twenty-five µl of the undiluted and serially diluted serum was mixed with 25µl of test virus containing four hemagglutination units in each well of a microplate and incubated at room temperature for 30 min. Then 50µl of 0.5% chicken erythrocytes was added to each well. Results were read after incubation at room temperature for 30 min. HI antibody titers were defined as the reciprocal of the highest serum dilution that prevented virus-mediated hemagglutination of the chicken erythrocytes.

References

1. World Health Organization. WHO Manual on Animal Influenza Diagnosis and Surveillance. 2002 [cited 2017 Jul 25]. http://www.who.int/csr/resources/publications/influenza/en/whocdscsrncs20025rev.pdf

2. Yu Z, Cheng K, Sun W, Xin Y, Cai J, Ma R, et al. Lowly pathogenic avian influenza (H9N2) infection in Plateau pika (Ochotona curzoniae), Qinghai Lake, China. Vet Microbiol. 2014;173:132–5. PubMed http://dx.doi.org/10.1016/j.vetmic.2014.07.002

Technical Appendix Table 1. Identification of viruses possessing gene segments with the highest nucleotide identity to each segment of A/black-tailed gull/Weihai/115/2016 (H13N2) and A/black-tailed gull/Weihai/17/2016 (H13N8) based on sequences available in GenBank*

Virus, gene	Virus with the highest percentage of nucleotide identity	GenBank accession no.	Identity, %
A/black-tailed gull/Weihai/115/2016 (H13N2)			
PB2	A/northern shoveler/Interior Alaska/1/2007(H12N5)	CY038351.1	94.2
PB1	A/duck/Shiga/8/2004(H4N6)	AB304145.1	96.6
PA	A/wild goose/Dongting/C1037/2011(H12N8)	KC876690.1	97.7
HA	A/black-headed gull/Netherlands/31/2009(H13N2)	KX979380.1	97.1
NP	A/black-headed gull/Netherlands/10/2012(H13N6)	MF148105.1	98.8
NA	A/duck/Hokkaido/WZ1/2014(H11N2)	LC042067.1	98.8
M	A/black-headed gull/Netherlands/31/2009(H13N2)	KX979549.1	98.1
NS	A/black-headed gull/Netherlands/4/2015(H16N3)	KX978185.1	99.2
A/black-tailed gull/Weihai/17/2016 (H13N8)			
PB2	A/northern shoveler/Interior Alaska/1/2007(H12N5)	CY038351.1	94.8
PB1	A/duck/Shiga/8/2004(H4N6)	AB304145.1	96.6
PA	A/wild goose/Dongting/C1037/2011(H12N8)	KC876690.1	97.6
HA	A/yellow-legged gull/Georgia/4/2012(H13N8)	MF147792.1	97.6
NP	A/black-headed gull/Netherlands/10/2012(H13N6))	MF148105.1	98.9
NA	A/black-headed gull/Netherlands/10/2013(H13N8)	KX978567.1	96.9
M	A/black-headed gull/Netherlands/10/2013(H13N8)	KX979140.1	99.3
NS	A/black-headed gull/Netherlands/4/2015(H16N3)	KX978185.1	99.2

*HA, hemagglutinin; M, matrix; NA, neuraminidase; NP, nucleoprotein; NS, nonstructural protein; PA, acidic polymerase PB, basic polymerase.

Technical Appendix Table 2. Analysis of molecular features associated with mammalian virulence, transmissibility, and antiviral resistance*

Protein	Molecular feature or amino acid substitution†	Phenotypic effect	A/black-tailed gull/Weihai/115/2016 (H13N2)	A/black-tailed gull/Weihai/17/2016 (H13N8)
PB2	E158G	Enhanced polymerase activity and increased virulence	E	E
	E627K	Enhanced polymerase activity and increased virulence	E	E
	D701N	Enhanced polymerase activity and increased virulence	D	D
PB1	H99Y	Associated with H5 transmissibility in ferrets	H	H
	I368V	Associated with H5 transmissibility in ferrets	I	I
PA	T97I	Enhanced polymerase activity and increased virulence	T	T
HA	Multibasic cleavage site	Expanded viral tropism; increased virulence in mice	Absent	Absent
	H107Y	Associated with H5 transmissibility in ferrets	H	H
	T160A	Associated with H5 transmissibility in ferrets	K	T
	Q226L	Human-type receptor binding; associated with H5 transmissibility in ferrets	Q	Q
	G228S	Human-type receptor binding; associated with H5 transmissibility in ferrets	S	S
NA	Stalk deletion	Increased virulence in mice	Absent; no deletion	Absent; no deletion
	H274Y	Oseltamivir resistance	H	H
	N294S	Oseltamivir resistance	N	N
M1	N30D	Increased virulence in mice	D	D
	T215A	Increased virulence in mice	A	A
M2	S31N	Amantadine resistance	S	S
NS1	P42S	Increased virulence in mice	S	S

*Single letters refer to the amino acid (aa) found in the noted gene at a specific site. HA, hemagglutinin; M, matrix; NA, neuraminidase; NP, nucleoprotein; NS, nonstructural protein; PA, acidic polymerase PB, basic polymerase. Underlining indicates molecular features that were associated with virulence in mammals or transmissibility of influenza virus and found in H13 isolates in this study.
†Sites are numbered from M, the start codon.

Technical Appendix Table 3. Chicken serum antibodies against subtype H13N2 virus A/black-tailed gull/Weihai/115/2016 (H13N2) or subtype H13N8 virus A/black-tailed gull/Weihai/17/2016 (H13N8)

	HI titers against viruses*	
Sample no.	H13N2	H13N8
3	10	<10
9	10	<10
12	10	<10
30	10	<10
1	<10	10
4	<10	20
7	<10	20
11	<10	20
18	<10	20
21	<10	20
22	<10	20
23	<10	20
24	<10	20
29	<10	20
34	<10	10
36	<10	20
46	<10	10
47	<10	10

*Only display the HI results of HI-positive serum samples against subtype H13N2 or H13N8 virus.

Technical Appendix Table 4. Primer pairs used for the PCR amplification of the HA and NA gene segment

HA or NA subtype	Primers (5′→3′)
H13	H13-F: AGCAAAAGCAGGGGAGAATTC<br>H13-R: AGTAGAAACAAGGGTGTTTTTTCTGC
H16	H16-F: AGCAAAAGCAGGGGATATTGTC<br>H16-R: AGTAGAAACAAGGGTTTTTTTCCG
N2	N2-F: AGCAAAAGCAGGAGTGAAAAT<br>N2-R: AGTAGAAACAAGGAGTTTTTTCTAA
N3	N3-F: AGCAAAAGCAGGTGTGAAAT<br>N3-R: AGTAGAAACAAGGTGTTTTTTCTAT
N6	N6-F: AGCAAAAGCAGGGTGAC<br>N6-R: AGTAGAAACAAGGGTGTTTTTC
N8	N8-F: AGCAAAAGCAGGAGTTTAAAAT<br>N8-R: AGTAGAAACAAGGAGTTTTTTCGT

Technical Appendix Table 5. Primer pairs used for the PCR amplification of the PB2, PB1, PA, NP, M, and NS gene segment of H13N2 and H13N6 viruses

Gene segment	Primers (5′→3′)
PB2	H13-PB2-F: AGCAAAAGCAGGTCAAATATAT<br>H13-PB2-R: AGTAGAAACAAGGTCGTTTTTAA
PB1	H13-PB1-F: AGCAAAAGCAGGCAAACCAT<br>H13-PB1-R: AGTAGAAACAAGGCATTTTTTCATG
PA	H13-PA-F: AGCAAAAGCAGGTACTGATC<br>H13-PA-R: AGTAGAAACAAGGTACTTTTTTGG
NP	H13-NP-F: AGCAAAAGCAGGGTAGATAATC<br>H13-NP-R: AGTAGAAACAAGGGTATTTTTCTTC
M	H13-M-F: AGCAAAAGCAGGTAGATA<br>H13-M-R: AGTAGAAACAAGGTAGTTTTT
NS	H13-NS-F: AGCAAAAGCAGGGTGACAA<br>H13-NS-R: AGTAGAAACAAGGGTGTTTTTTATC

Technical Appendix Table 6. Hemagglutination inhibition antibody titers of reference serum sample against influenza viruses of different subtypes*

Antigens†	H1	H3	H4	H5	H6	H7	H9	H13N2	H13N8
H1	160	<10	<10	<10	<10	<10	<10	<10	<10
H3	<10	160	<10	<10	<10	<10	<10	<10	<10
H4	<10	<10	160	<10	<10	<10	<10	<10	<10
H5	<10	<10	<10	80	<10	<10	<10	<10	<10
H6	<10	<10	<10	<10	80	<10	<10	<10	<10
H7	<10	<10	<10	<10	<10	80	<10	<10	<10
H9	<10	<10	<10	<10	<10	<10	80	<10	<10
H13N2	<10	<10	<10	<10	<10	<10	<10	80	<10
H13N8	<10	<10	<10	<10	<10	<10	<10	<10	80

*Nine reference serum samples were used in hemagglutination inhibiting serum antibody titers tests. The reference serum samples for H1, H3, H4, H5, H6, H7, and H9 subtype avian influenza viruses were collected from specific pathogen-free chickens and provided by the Military Veterinary Research Institute of Academy of Military Medical Sciences. The reference serum samples for H13N2 and H13N8 subtype avian influenza viruses were collected from specific pathogen-free chickens by our laboratory.

†Nine influenza virus subtypes were used in hemagglutination inhibiting serum antibody titers tests: H1 (A/black-necked crane/Zhaotong/ZT-12/2013 [H1N2]), H3 (A/baikal teal/Shanghai/SH-89/2013 [H3N2]), H4 (A/greylag goose/Changsha/CS-510/2013 [H4N8]), H5 (A/great tit/Panjing/PJ-66/2013 [H5N1]), H6 (A/common teal/Nanji/NJ-227/2013 [H6N1]), H7 (A/Baer's pochard/HuNan/414/2010 [H7N1]), H9 (A/great bustard/InnerMongolia/IM-E2/2012 [H9N2]), H13N2 (A/black-tailed gull/Weihai/115/2016 [H13N2]), and H13N8 (A/black-tailed gull/Weihai/17/2016 [H13N8]).

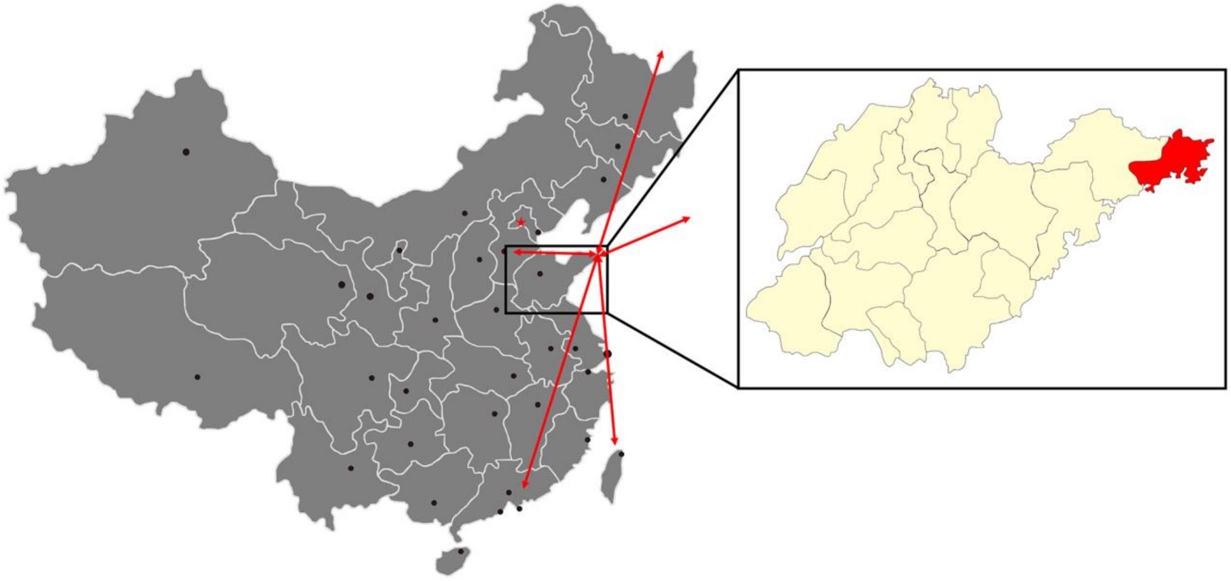

Technical Appendix Figure 1. The migratory routes of the black-tailed gulls (*Larus crassirostris*) to Weihai city, Shandong province, China. Red color area indicates Weihai city. Arrows indicate the migratory routes.

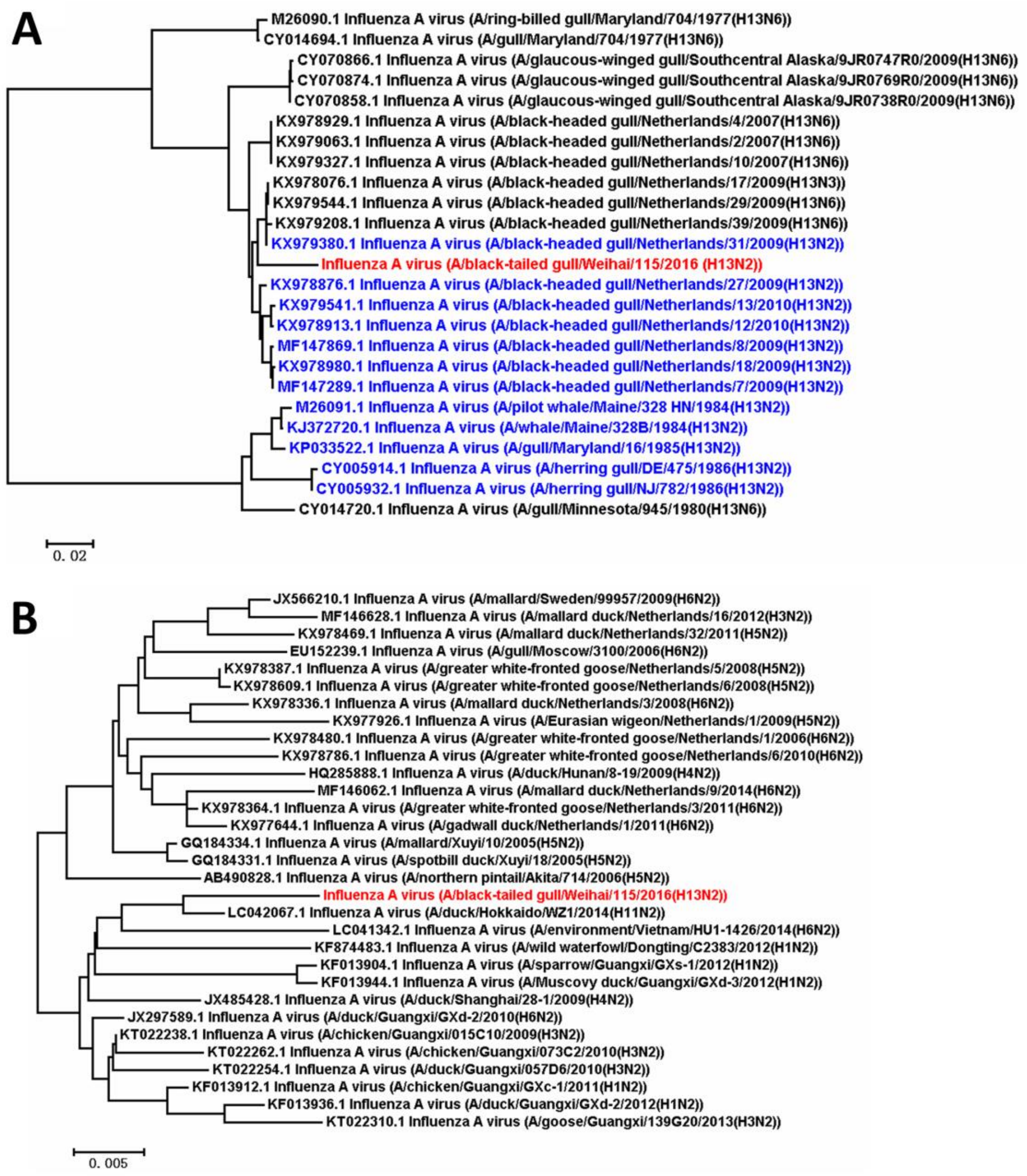

Technical Appendix Figure 2. Phylogenetic trees of HA (A) and NA (B) genes of novel avian influenza (H13N2) virus isolated from black-tailed gull in China, December 2016, with reference sequences. Red, A/black-tailed gull/Weihai/115/2016 (H13N2); blue, other H13N2 avian influenza viruses. Trees were generated by applying the neighbor-joining method in MEGA 7.0 (www.megasoftware.net) on the basis of full-length sequences. Scale bars indicate branch length based on number of nucleotide substitutions per site.

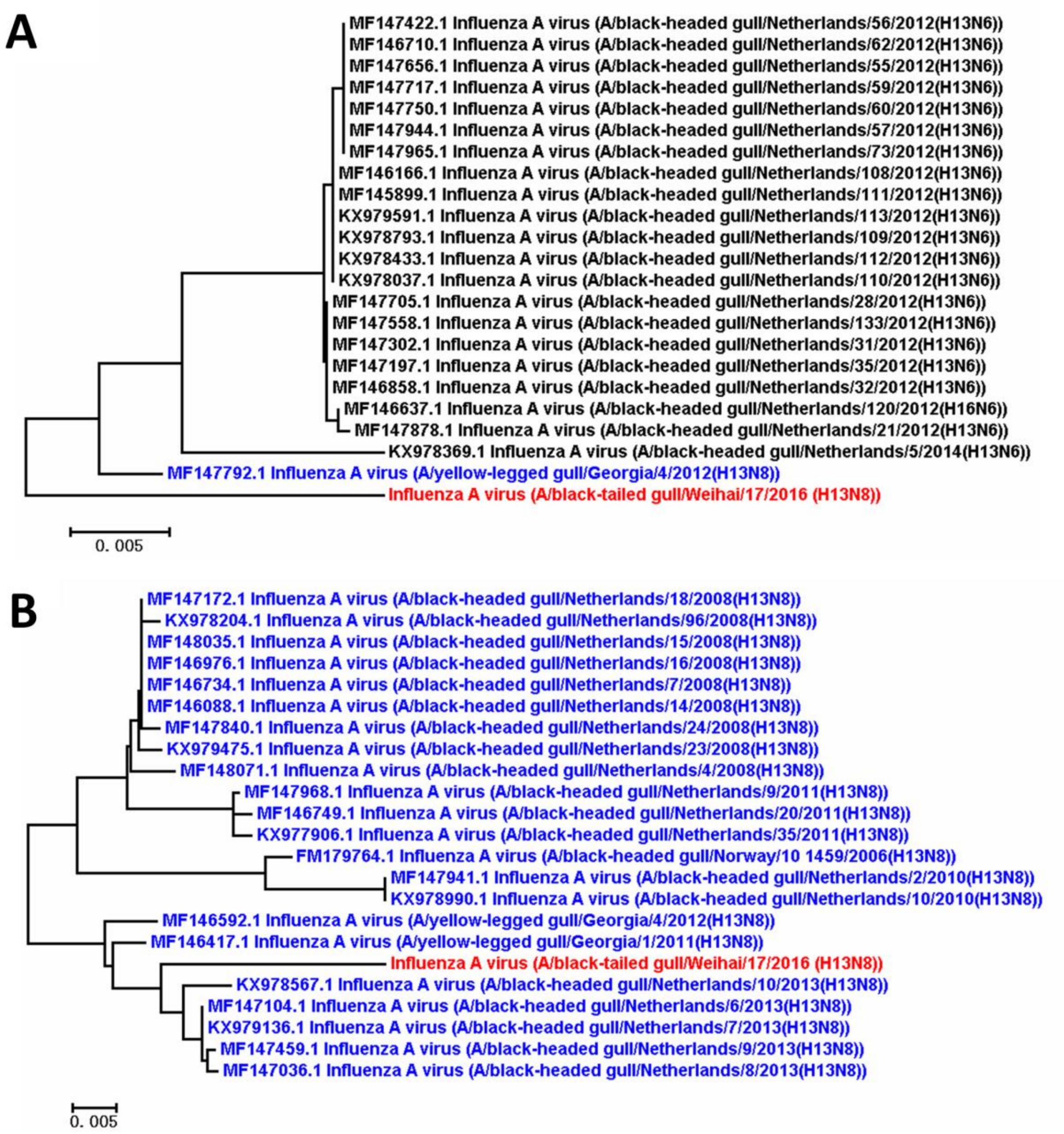

Technical Appendix Figure 3. Phylogenetic trees of HA (A) and NA (B) genes of novel avian influenza (H13N8) virus isolated from black-tailed gull in China, December 2016, with reference sequences. Red, A/black-tailed gull/Weihai/17/2016 (H13N8); blue, other H13N8 avian influenza viruses. Trees were generated by applying the neighbor-joining method in MEGA 7.0 (www.megasoftware.net) on the basis of full-length sequences. Scale bars indicate branch length based on number of nucleotide substitutions per site.

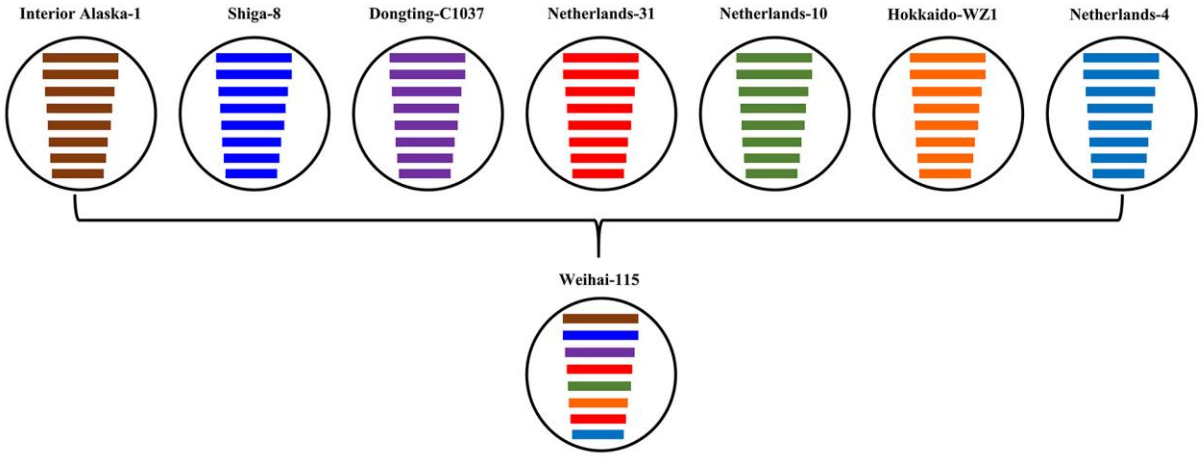

Technical Appendix Figure 4. Putative genomic compositions of the novel avian influenza (H13N2) virus isolated from black-tailed gull in China, December 2016, with their possible donors. The 8 gene segments (from top to bottom) in each virus are polymerase basic protein 2 (PB2), polymerase basic protein 1 (PB1), polymerase acidic protein (PA), hemagglutinin (HA), Nucleoprotein (NP), neuraminidase (NA), matrix protein (M), and nonstructural protein (NS). Each color indicates a separate virus background. Interior Alaska-1, A/northern shoveler/Interior Alaska/1/2007(H12N5); Shiga-8, A/duck/Shiga/8/2004(H4N6); Dongting-C1037, A/wild goose/Dongting/C1037/2011(H12N8); Netherlands-31, A/black-headed gull/Netherlands/31/2009(H13N2); Netherlands-10, A/black-headed gull/Netherlands/10/2012(H13N6); Hokkaido-WZ1, A/duck/Hokkaido/WZ1/2014(H11N2); Netherlands-4, A/black-headed gull/Netherlands/4/2015(H16N3). The simplified schematic illustration is based on nucleotide distance comparison and phylogenetic analysis.

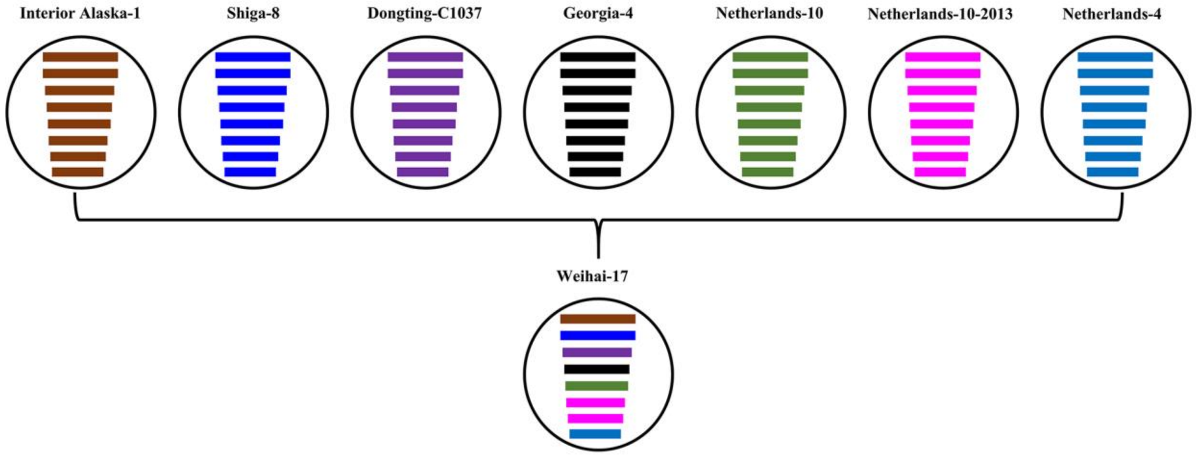

Technical Appendix Figure 5. Putative genomic compositions of the novel avian influenza (H13N8) virus isolated from black-tailed gull in China, December 2016, with their possible donors. The 8 gene segments (from top to bottom) in each virus are polymerase basic protein 2 (PB2), polymerase basic protein 1 (PB1), polymerase acidic protein (PA), hemagglutinin (HA), Nucleoprotein (NP), neuraminidase (NA), matrix protein (M), and nonstructural protein (NS). Each color indicates a separate virus background. Interior Alaska-1, A/northern shoveler/Interior Alaska/1/2007(H12N5); Shiga-8, A/duck/Shiga/8/2004(H4N6); Dongting-C1037, A/wild goose/Dongting/C1037/2011(H12N8); Georgia-4, A/yellow-legged gull/Georgia/4/2012(H13N8); Netherlands-10, A/black-headed gull/Netherlands/10/2012(H13N6); Netherlands-10–2013, A/black-headed gull/Netherlands/10/2013(H13N8); Netherlands-4, A/black-headed gull/Netherlands/4/2015(H16N3). The simplified schematic illustration is based on nucleotide distance comparison and phylogenetic analysis.
